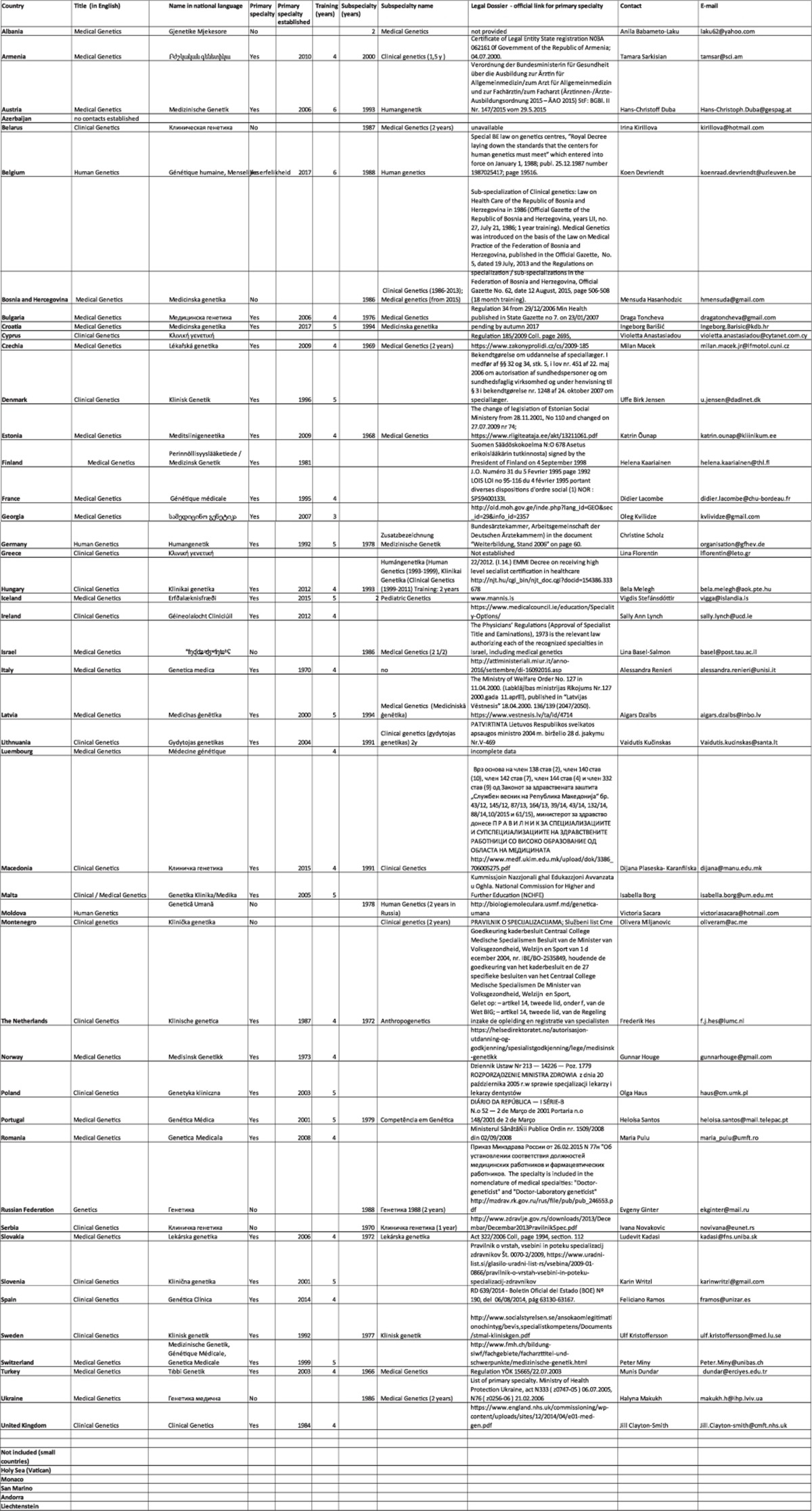# From Mendel to Medical Genetics

**DOI:** 10.1038/ejhg.2017.157

**Published:** 2018-01-03

**Authors:** Ulf Kristoffersson, Milan Macek

**Affiliations:** 1 Department of Clinical Genetics and Pathology, Lund University, Lund University Hospital, Lund, Sweden; 2 Charles University and Motol University Hospital, Prague, Czech Republic

## Introduction

Only a few years after the rediscovery of Mendel’s laws of inheritance in 1900, the first human genetic disorders and variants were described.^1, 2^ Although these disorders were considered to be rare exceptions, the number of disorders to follow a Mendelian pattern of inheritance increased slowly. Victor McKusick, in his first volume of Mendelian Inheritance in Man published in 1966, listed 1486 entries, mainly phenotypes, whereas the current online catalogues contain over 8000 entries, with more than 5000 with a known molecular basis.^3, 4, 5^

The discovery of DNA as a carrier of the genetic code, its double helix structure and the rapidly developing possibility of the clinical use of chromosome- and DNA analyses made expertise in medical genetics (MG) valuable in health-care services and a subspecialty started to grow, mainly in gynaecology, neurology, paediatrics and laboratory medicine.

With the birth of the European Union (EU), a need for collaboration between established specialities emerged and the Union of European Medical Specialists (UEMS)^6^ was founded in the same year that the Treaty of Rome^7^ was signed. UEMS is an association of national medical professional organisations which focus on the harmonisation of training and education of medical doctors within and across all medical specialties. A number of specialities were soon mutually recognised in the member states as equivalent in training, leading to a national speciality licence being mutually recognised in all member states. At this time, MG was not recognised in any European country.

In this article, which includes the results of a recent survey, we describe the development of MG as a specialty in all European countries – not only within the EU itself – and the process undertaken in order to acquire its de iure recognition in the EU.

## The process to European union recognition

The youthful status of our speciality is reflected in the fact that it has different names in different countries – MG, clinical genetics and human genetics being the most common, as they appear in the current version of European Directive 2005/36/EC on the recognition of professional qualifications^8^ (Professional Qualifications Directive; PQD). In this paper, we will refer to MG.

The process of becoming an EU-recognised speciality started with a discussion in the European Society of Human Genetics (ESHG) Public and Professional Policy committee shortly after it was founded in 1997, but it was too late to have MG included in the 1999 revision of the PQD.

At the ESHG board meeting in Munich 2004, Jean-Jacques Cassiman brought up the issue again, as he had met with the Secretary General of UEMS. After a discussion in the ESHG board, Ulf Kristoffersson was appointed to lead an *ad hoc* committee together with Dian Donnai, who were later replaced by Helen Kingston and Didier Lacombe. Their task was to draft common European guidelines for medical training with a specialisation in MG. After 2 years, we were finished and the document was endorsed by the ESHG membership.

Important support came with the adoption of the Organisation for Economic Co-operation and Development (OECD) ‘Guidelines for quality assurance in molecular genetic testing’ (2007),^9^ where many members of ESHG and the EuroGentest Network of Excellence EU project^10^ were involved in the drafting. Article E5 of the Guidelines stipulates that ‘Relevant government or professional authorities should recognise MG as a discipline comprising both a clinical and a laboratory specialty,’ thus underlining the multidisciplinary character of genetic services and the need for official recognition of the medical and clinical laboratory professional branches involved in the provision of genetic services (see later). Further significant backing for the recognition of MG emerged when, in May 2008, the first international legally binding instrument concerning genetic testing for health purposes was adopted by the Committee of Ministers of the Council of Europe.^11^

In parallel, we established contact with UEMS and initiated the procedures necessary for us to become a member organisation representing MG. Only recognised specialities could become ‘sections’, but if at least two recognised specialities so wished, a Multidisciplinary Joint Committee (MJC) could be formed. Thus, with help of the sections of Paediatrics and Obstetrics and Gynaecology, an MJC for ‘Clinical Genetics’ was formed according to the procedures and statutes of UEMS. Ulf Kristoffersson was elected the Chair and Helen Kingston the Secretary. Being an MJC, we received a voice in UEMS and afterwards the UEMS council also adopted our ESHG-approved training guidelines as UEMS guidelines: ‘Description of Clinical Genetics as a medical specialty in the EU: Aims and objectives for specialist training’ (April 2009; amended 2017).^12^

During the European Human Genetics Conference in Vienna on 25 May 2009, the vast majority of those attending the 5th Meeting of the Presidents of the National Human Genetics Societies (NHGS)^13^ signed a joint petition in support of the inclusion of MG in the PQD and endorsed the aforementioned UEMS consensus training curriculum. In addition, Jean-Jacques Cassiman contacted Frieda Brepoels, one of the Belgian members of the EU Parliament. She proposed a vote in favour of the recognition of MG in a Parliament Committee in March 2009. Unfortunately, there were not enough votes in favour for the motion to be carried.

Another important boost for the recognition of MG came from the successive French and Czech EU Council presidencies of the EU in July–December 2008 and January–June 2009, respectively. In November 2008, during the French term, John Burn and Arnold Munnich visited the French Minister of Health, Roselyne Bachelot, and asked for France to issue a formal request to the EC to start recognition proceedings. Indeed, the ‘French Request for inclusion of the specialty of MG under Annex V’ into PQD was later officially filed, following additional support from French Orphanet representatives (Ségolène Aymé) in March 2009. Concurrently, Milan Macek was the chief government advisor to the Czech Presidency. He worked closely with EURORDIS-Rare Diseases Europe, a non-profit alliance of over 700 European rare disease (RD) patient organisations (represented by Yann le Cam and his team) for the passage of the ‘EU Council on Recommendation on an action in the field of RD (2009/C151/02).^14^ After intensive work at the Council and lobbying within the 6-month window of opportunity, this key EU document was adopted in June 2009.

The provisions of the Council Recommendation created a strong momentum for the recognition of MG by setting out the relevance of training in the specialty for the diagnosis of RD, of which over 80% are genetic. Moreover, its Recital 15 provided us with justification for the cross-border mobility of MG (ie, ‘expertise should travel rather than patients themselves’), it being the first line of diagnostic contact for the majority of these disorders. This clause was particularly relevant, since the PQD lists only those specialties where there is a justified need for cross-border provision of medical care and where there is a ‘bottom up’ consensus on a given postgraduate training curriculum by EU Member States for a particular medical specialty, that is, via the UEMS.

Following the French request to the EC, the ESHG worked with the NHGS representatives in providing the Recognition Committee (RC), an official EC body formed of member state representatives that has the power to authorise the EC to amend the PQD. At that time, EU presidents provided their national representatives at this committee with (a) endorsements of the UEMS consensus MG curriculum, including the harmonisation of respective national MG curricula with UEMS provisions and a minimal duration of postgraduate training of 4 years, (b) where applicable, legal dossiers stipulating national recognition of MG in their own countries and thus (c) ‘evidence-based’ support letters for the European recognition of MG.^15^ These activities were coordinated by Milan Macek, who at that time served as the President of the ESHG, and were spearheaded by the Czech RC representative (Lucia Slobodová). By mid-2010, the RC was provided with the official evidence that MG is recognised as a medical specialty at the national level in 20 of the 27 EU member states, that is, as a primary specialty termed 8 × ‘clinical-’, 10 × ‘medical-’, 1 × ‘human-’ and 1 × genetics, while in Hungary MG was a subspecialty at that time. This overall number of national recognitions was greater than the qualified majority needed for a decisive vote by the RC (October 2010). Finally, on 3 March 2011, the EC adopted ‘Regulation (EU) No 213/2011 amending Annexes II and V to Directive 2005/36/EC of the European Parliament and of the Council on the recognition of professional qualifications’.^16^ This administrative act means that MG is now officially recognised as a European specialty. Subsequently, EU recognition of MG facilitated national recognitions in Spain (2014), Belgium and Croatia (both 2017), the transition of MG to a primary specialty status in Hungary (2012) and the creation of a new professional society in Iceland (2012).

After the EU recognition we applied, with the help of the Swedish Medical Association, to form a Section for Clinical Genetics^17^ which was approved by the UEMS Council in 2013. Ulf Kristoffersson was elected the first president, and was followed by Bela Melegh in 2015. The three main tasks have been to update the training guidelines, to draft a syllabus for training and to develop a protocol for an European specialist exam planned to be offered for the first time in 2018. In 2016, the Section took the initiative of forming a ‘MJC for Rare and Undiagnosed Disorders (MJC-RUD)’, in order to be able to form a bridge between the UEMS and the newly established European Reference Networks (ERN) for RD^18^ for collaboration on the harmonisation of MG training and education.

## The development of medical genetics in Europe

The 1997 survey ‘Medical Genetics in Europe’ provided evidence that 15 of the 24 EU countries participating in this exercise recognised MG.^19^ In the spring of 2017, we performed an update of national legislative documents regulating MG in all member states of the Council of Europe,^20^ 47 in all, adding Belarus as the only European country not being a member of this international organisation, and Israel, which is an ‘Observer to the Parliamentary Assembly’. Five minor member states were not included in this survey (Andorra, Lichtenstein, Monaco, San Marino and the Vatican) as they usually utilise the provisions and/or genetic services of their neighbouring countries. From one country, Azerbaijan, no information was available (Table 1) and for Luxembourg information was drawn from the data listed in the PQD. In Table 2 aggregated data on the year of recognition of specialisation and/or subspecialisation is presented, and in Table 3 the length of training is summarised (data drawn from the Table 4).

## Discussion

At present all but two European countries, Greece and Cyprus, have recognised the MG speciality; Belgium and Croatia as late as this year. Seventeen countries recognised MG as a subspecialty before 2000, and nine of them later changed the status to a stand-alone, that is, primary, speciality. This recognition went slowly until the turn of the millennium, when the scientific progress in human molecular genetics made the discipline an important partner in the development of health care and further evolved with the concept of personalised (stratified or precision) medicine. Full recognition was adopted in 21 countries, that is, about half of the Council of Europe member states, after 2000 (see above).

Training requirements for specialisation varies between the countries ranges from 3 to 6 years, the most common duration being 4 years which is also the minimum length stipulated by PQD. The content of training varies between countries, especially regarding the amount of laboratory competence needed and requirement of clinical electives in other related medical specialties (eg, gynaecology, neurology and/or paediatrics). In spite of the different languages and the varying tasks of a specialist in MG in different European countries, we have now the possibility of working in many different settings and environments, an opportunity that we hope many young doctors will take advantage of.

MG also aims to collaborate closely with the two other professional branches involved in genetic services, clinical laboratory geneticists and genetic nurses and counsellors, under the auspices of the European Board of Medical Genetics (EBMG).^21^ This independent board was established in 2012 to serve the needs of patients through establishing standards of practice in all professional branches providing genetic services, and to ultimately issue professional certifications.

Finally, recognition of MG will also aid implementation of Articles 54 and 55 of Directive 2011/24/EU of the European Parliament and of the Council on the application of patients’ rights in cross-border healthcare,^22^ which provide special provisions for RD and was seminal for the development of ERNs, where MG is embedded as a core specialty in the majority of their cross-border, interdisciplinary research and diagnostic activities.

## Figures and Tables

**Table 1:** Current status of the medical genetics specialty in Europe: results of a 2017 survey

*No of countries in the Council of Europe*	*47*
Belarus added	1
Countries not included	4 (Monaco, Andorra, San Marino, Lichtenstein, Vatican)
No or incomplete data	2 (Azerbaijan and Luxembourg)
Countries included	42
No established specialty	2 (Greece and Cyprus)
No specialty but subspecialty	8
Currently primary specialty	32
Subspecialty before 1997	17
Primary specialty before 1997	15

**Table 2:** Agregated data on the year of recognition of medical genetics specialisation and/or subspecialisation

*Year for specialty recognised*	*Subspecialty recognised*	*Still subspecialty*	*Before specialty*
XXXX–1975	2	4	1
1976–1980	0	4	2
1981–1985	2	0	0
1986–1990	1	2	3
1991–1995	3	6	0
1996–2000	3	1	0
2001–2005	5	0	0
2006–2010	8	0	0
2011–2015	6	0	0
2016–2017	2	0	0
Sum	30	17	6
No data	2		

**Table 3:** Length of postgraduate training in the medical genetics specialty

*Years of training for primary specialisation*	
3 years	1
4 years	16
5 years	8
6 years	2
Missing data	5
Sum	32

**Table 4:**